# Parents’ perspectives of the new neonatal BCG vaccination pathway in England: a qualitative study

**DOI:** 10.1186/s12889-025-23859-x

**Published:** 2025-08-18

**Authors:** Kate Bisset, Georgia Chisnall, Colin N.J. Campbell, Vanessa Saliba, Sandra Mounier-Jack, Tracey Chantler

**Affiliations:** 1 London School of Hygiene and Tropical Medicine, London, UK; 2 UK Health Security Agency, London, UK

**Keywords:** Neonatal BCG, Tuberculosis, Vaccine uptake, Parental experiences, SCID screening, England

## Abstract

**Background:**

The neonatal Bacillus Calmette-Guérin (BCG) selective vaccination pathway in England was revised in September 2021 due to the introduction of a national evaluation of newborn screening for Severe Combined Immunodeficiency (SCID). BCG is a live attenuated vaccine that is contraindicated in infants with SCID, hence BCG vaccination was moved from soon after birth to after SCID results were available, typically at 14–17 days. The transition also shifted vaccination delivery from maternity units to community clinics, raising concerns about potential barriers to access and lower vaccine uptake.

This study explored parents’ experiences of navigating the new neonatal BCG vaccination pathway and identified access barriers and enablers.

**Methods:**

A qualitative study was conducted involving semi-structured interviews with 30 parents of infants eligible (or invited) for BCG vaccination in two urban areas where SCID screening was implemented. Participants were recruited through vaccine providers and community centres. Thematic analysis of interview transcripts was conducted using the ‘Framework Method’, incorporating an inductive approach.

**Results:**

Parents were unaware of SCID screening and the changes to the neonatal BCG vaccination schedule and encountered diverse challenges in accessing the vaccine. Assessment errors led to eligibility confusion, with some ineligible infants receiving vaccine invitations. Many parents first learned about BCG vaccination on the postnatal ward, describing it as a “surprise vaccine,” with limited antenatal discussion diminishing informed decision-making. Appointment notification systems were inconsistent, with some parents receiving short-notice invitations or no notification at all. Physical access barriers included unfamiliar and distant clinic locations, transport, and the physical challenges of traveling soon after birth with a newborn. Parents with limited social support or financial constraints faced additional difficulties.

**Conclusion:**

Parents were generally accepting of the need to amend the BCG timeline on account of SCID screening; however, we identified distinct accessibility concerns that varied from those associated with the routine immunisation programme. These barriers, and the separateness of the BCG programme from routine services, impacted parental experiences and vaccine uptake. Addressing these challenges is important to meet neonatal BCG uptake targets and support tuberculosis prevention efforts in England.

**Supplementary Information:**

The online version contains supplementary material available at 10.1186/s12889-025-23859-x.

## Introduction

The Bacillus Calmette-Guerin (BCG) vaccine has been part of the prevention of tuberculosis (TB) in England since 1953 when it was first given to 14-year-olds as part of a universal school immunisation programme [1]. Since then, several changes were made to the BCG programme due to the changing epidemiology of TB in England, as documented in The Green Book (a UK government resource on vaccines and vaccination procedures) [1]. Currently the BCG vaccine is offered as a selective immunisation programme for those at highest risk of contracting TB. A key component of the programme is the selective neonatal BCG programme which protects babies most at risk of exposure to TB, who are more vulnerable to serious forms of TB such as TB meningitis [2].

Neonatal BCG Eligibility criteria:

- parent/s or grandparent/s being born in a country where the annual incidence of TB is 40/100,000 or greater.
- living in areas of England where the annual incidence of TB is 40/100,000 or greater [3].

The neonatal BCG vaccination pathway was revised in September 2021, following the introduction of national evaluation of screening for Severe Combined Immunodeficiency syndrome (SCID) [4]. SCID is a rare inherited condition caused by mutations to genes responsible for the development of T-lymphocytes [5] and if untreated can result in death due to impairment of the immune system. SCID was added to the list of diseases screened for in the newborn blood spot (NBS) test carried out 5 days after birth [6] as part of a national evaluation conducted in six areas (Manchester, Birmingham, Sheffield, Newcastle, London Great Ormond Street Hospital and London Southeast Thames), covering two thirds of newborns in England [7]. Live vaccines, including the BCG vaccine that contains a live attenuated strain derived from M. Bovis, are contraindicated in infants who have SCID [8]. This is due to the increased risk of adverse events from the vaccine such as disseminated BCG disease (BCGosis) [9].

In October 2018 the Joint Committee for Vaccination and Immunisation (JCVI) advised that the BCG vaccine should be moved from ‘soon after birth’, to ‘after SCID results were available’ (usually 14–17 days after birth) to prevent those who screen positive for SCID receiving a BCG vaccine [10]. The new BCG pathway aims to vaccinate eligible babies by 28 days (or soon after), to ensure early protection from TB, but babies can have the vaccination earlier if the SCID screening result is available [10]. The change was applied nationally, not just in the six pilot evaluation areas, to ensure consistency and safety [7]. For many areas in England this meant moving the BCG vaccine delivery away from maternity units, where it was often administered while the baby was still in hospital, to a community provider and venue [10, 11].

Stakeholders, including neonatologists and paediatricians, raised concerns that a community clinic delivery model may reduce uptake compared with the previous maternity model [10, 11]. Parents of eligible babies’ mother tongue may not be English, and some could be members of underserved communities, who may experience difficulties navigating and accessing NHS services [11–13]. These barriers could result in a decline in vaccine coverage leaving eligible babies unprotected and at risk of developing TB in infancy and beyond. JCVI recommended that the new community delivery model for BCG vaccine needed to be as robust as the previous hospital bedside delivery model [10].

The UK Health Security Agency (UKHSA) and NHS England remain committed to meeting World Health Organisation (WHO) TB elimination targets and have developed a TB action plan [14]. While England remains a low TB incidence country (≤ 10 per 100,000), most recent epidemiology shows 4,855 people were notified to UKHSA with TB in 2023, which is an 11% increase from 2022. In children, there were 259 notifications in 2023, which is an increase of 12% from 2022 [15]. Ensuring the effective delivery of the BCG programme, including offering BCG to 100% of those who are eligible and achieving 80% uptake, are integral components of TB prevention in England [14].

This study follows a service evaluation published in 2024 that explored the experience of commissioners and providers tasked with implementing the new BCG pathway [16]. The evaluation identified challenges associated with ensuring eligible babies were vaccinated by 28 days, including increased Did Not Attend^1^ (DNA) rates, challenging data and appointment systems, and insufficient staffing and resourcing for the new delivery model. Fifty-two per cent of providers reported DNA rates of 20–59%. Commissioners and providers were unsure, or could only hypothesise, about the reasons for increased DNA rates or any issues parents had with navigating the BCG system.

Drawing on their personal experiences of service delivery they cited the following suspected barriers: English literacy; demographic-related health inequalities; clinic suitability; concern about how young their babies were and perceived proximity of the BCG vaccine to the routine vaccinations offered at 8-weeks of age; and a desire for more time to make decisions. Given the changes to the programme 74% of providers suggested that improving BCG vaccine health education was vital. Despite these concerns, only 44% of providers reported having completed an accessibility assessment at the point of interview (more than one year into the revised service delivery) [16]. Furthermore, most clinics did not offer evening or weekend services (76%) and reported travel distances of up to 10 miles (57%). Some clinics described bespoke initiatives to address DNA and access constraints, but these required additional human and financial resources which were not available to all BCG service providers.

These findings indicated the need to examine parents’ experiences navigating the new pathway to inform appropriate service delivery improvements and by extension improve vaccine uptake. The research reported in this paper aimed to understand parents’ experiences and the enablers and barriers they face in accessing the BCG vaccine in the new model of delivery.

## Methods

### Design

To evaluate and explore parents with infants aged ≤ 1 year who met the neonatal BCG vaccine eligibility criteria or received a BCG vaccination invitation experience of the BCG vaccination pathway we followed an observational qualitative study design. Semi-structured interviews (SSIs) were conducted with parents between September 2023 and January 2024.

### Sampling and recruitment

Two urban areas, which were SCID screening evaluation sites, were selected to ensure parents would have experienced the new pathway in its entirety. These two areas matched the sites involved in providers and commissioners’ evaluation [16]. The locations are anonymised to maintain the confidentiality providers and commissioners as the number of staff overseeing the BCG pathway change in each area is small [16].

BCG vaccination providers supported recruitment by disseminating study information letters to parents when they sent out BCG vaccine invitation letters, or during BCG appointments. Community recruitment took place in community centres such as children’s centres where posters and flyers were put up and researchers attended “stay and play” sessions to discuss the study. These centres were located in areas with varying sociodemographic characteristics to help increase the representatives of our sample. Parents were asked to self-report eligibility in line with the eligibility criteria described in Table 1.

**Table 1:** Study eligibility criteria and rationale

Eligibility Criteria	Rationale
Under one year of age and born within the study setting (one of the two urban areas)	So that the oldest eligible infant would have completed the pathway after September 2022. At this point the new neonatal pathway would have been in place for at least one year, allowing us to explore experiences that should not reflect initial ‘teething’ issues with the new pathway in the early stages of implementation.
Be eligible OR received an invitation for BCG vaccination	Initially the eligibility criteria only specified infants who were ‘eligible’ for the BCG vaccine, however it became apparent in initial interviews that some of the infants who had received the vaccine did not meet the official eligibility criteria. This occurred after being referred by a healthcare worker or receiving an automated invitation for vaccination. This phenomenon is explored further in the results and discussion section.

Potential participants were given the opportunity to ask questions about the study before deciding to take part. Written informed consent was given by all participants prior to SSIs, which were conducted by London School of Hygiene and Tropical Medicine (LSHTM) researchers (KB, GC, TC). Ethical approval was granted by the UKHSA Research Support and Governance Office (Ref: NR0328).

### Data collection

SSIs were conducted online (via MS Teams or Zoom) or face-to-face in participants homes depending on their preference. The interview topic guide (Table S1) was developed with support of the conceptual framework depicted in Table 2 [17]. This framework posits that vaccine uptake is dependent on five factors spanning access, affordability, awareness, acceptance, and activation, defined in Table 2. Each area of the framework informed question development within the devised topic guide.

**Table 2:** The 5As taxonomy for determinants of vaccine uptake

Root cause	Definition	Contributing factors
1. Access	The ability of individuals to be reached by, or to reach, recommended vaccines	1.1 Place of birth 1.2 Location of vaccination 1.3 Contact with healthcare system 1.4 Convenience of access
2. Affordability	The ability of individuals to afford vaccination, both in terms of financial and non-financial cost (e.g., time)	2.1 Financial incentives 2.2 Time costs
3. Awareness	The degree to which individuals have knowledge of the need for, and availability of, recommended vaccines and their objective benefits and risks	3.1 Knowledge of vaccines and vaccination schedule 3.2 Availability of information 3.3 Consideration of vaccination
4. Acceptance	The degree to which individuals accept, question or refuse vaccination	4.1 Vaccine 4.1.1 Perceived safety 4.1.2 Perceived efficacy 4.1.3 Attitude to valence 4.2 Disease 4.2.1 Perceived severity 4.2.2 Vulnerability to risk 4.3 Individual characteristics 4.3.1 Health beliefs 4.3.2 Omission bias 4.3.3 Trust 4.3.4 Past behaviour 4.4 Social context 4.4.1 Social responsibility 4.4.2 Peer influence 4.4.3 HCW influence
5. Activation	The degree to which individuals are nudged towards vaccination uptake	5.1 Prompts and reminders 5.2 Workplace policies

Adapted from Thomson et al. (2016) [17]

Interviews were audio recorded and transcribed by a company that had signed a confidentiality agreement. Interview data was collected on encrypted, and password protected computers and recorders and stored (in compliance with the 2018 Data Protection Act) in a secure LSHTM data storage folder, that only LSHTM researchers (KB, TC, GC) could access via a double authentication process.

### Data analysis

The dataset was analysed using the ‘Framework Method’, a form of thematic analysis [18]. This approach was selected due to its systematic approach which fosters consistency in team approaches to analysis. This method consists of seven core stages: transcription, familiarization, coding, analytical framework development, application of the analytical framework to the transcripts, charting data into a framework matrix, and interpretation [18].

While a theoretical framework was used to design the SSI topic guide, an inductive approach was used for the analysis. This was appropriate due to the exploratory nature of the analysis; whereby key themes were constructed from the dataset without having to conform to a pre-devised analytical structure. This is a common approach for exploratory analysis and reduces the risk of analytical foreclosure or skewing of the findings [19, 20]. Using a blended inductive-deductive approach across data collection and analysis enabled us to theoretically ground our research and ensure we were asking the right questions while retaining the analytical freedom to present themes which were authentic to the original dataset.

Open coding was conducted independently by two researchers on five papers and used to generate a code book hosted in MS Excel (KB, GC). These codes where used to build the analytical framework into NVIVO 12 (qualitative analysis software produced by Lumivero) and applied to all transcripts (KB, GC, TC).

## Results

### Participants

Within our sample (*n* = 30) 16 babies received the BCG vaccine within 28 days of birth (as per the delivery target), 10 received it after 28 days, and four were not vaccinated. Of the 10 infants who were vaccinated after 28 days, four delays were due to parent choice and six to administrative issues. Within our sample, five infants were not eligible for BCG but received it because their parents had either been advised by a health care worker to get the vaccine or received an appointment notification (letter or text message). Participant characteristics are presented in Table 3.

**Table 3:** Summary of participant characteristics

Gender	Female (*n* = 25); Male (*n* = 5)
Age range*	25–40 years old
Location	Urban area 1 (*n* = 16); urban area 2 (*n* = 14)`
Vaccination status of infant**	Vaccinated ≤ 28days (*n* = 16) Vaccinated > 28 days old (*n* = 10) Of which: - due to admin issues (*n* = 6) - due to parent choice (*n* = 4) Not vaccinated (*n* = 4) Of which - Want to vaccinate infant (*n* = 2) - Chose not to vaccinate infant (*n* = 2)
Baby eligible for BCG	Yes (*n* = 25); No (*n* = 5)
Birth ranking of infant	First child (*n* = 13); Second (*n* = 10); Third/plus (*n* = 7)
Heritage of both parents as described by parent participant in their own words***	Albanian (*n* = 1); Australian (*n* = 1); Bangladeshi (*n* = 3); Brazilian (*n* = 2); Egyptian (*n* = 1); Ghanaian (*n* = 1); Indian (*n* = 6); Indian/East African(*n* = 1); Iranian (*n* = 1); Iraqi (*n* = 3); Kenyan (*n* = 1); Lithuania (*n* = 1); Nigerian (*n* = 4); North African (*n* = 1); Pakistani (*n* = 6); Romanian (*n* = 1); South Korean (*n* = 1); Sri Lankan (*n* = 1); Ukrainian (*n* = 1); West African (*n* = 1); White British (*n* = 4); Zimbabwean (*n* = 2); Unassigned (*n* = 16)
Migrant status for both parents***	First generation(*n* = 22); Second generation (*n* = 20); Not applicable (UK national) (*n* = 3); Unassigned (*n* = 15)

* Where own age disclosed by parent participant

**The target for the new neonatal BCG programme is to vaccinate eligible babies by 28 days old. For the purpose of this paper ‘delay’ is defined as vaccinated later than this target date.

***This includes both parents of the infant, not just the parent who was interviewed. This was recorded when freely offered by participants during interviews, or if needed to check for BCG eligibility

### Thematic findings

Our analysis identified several themes relating to enablers and barriers parents faced in accessing neonatal BCG vaccination. These themes are presented chronologically as the parents would encounter them on the vaccine pathway, from when they were first made aware of BCG, through to the appointment itself. An overview of themes and sub-themes are presented in Table 4.

**Table 4:** Core themes identified from parent interviews

Theme	Subtheme
1. Eligibility assessment gone wrong: incorrect referrals, confusion, and parental frustration	Assessment errors
	Parent understanding of risk and eligibility
2. How BCG information is provided by the health service	The ‘surprise’ vaccine
	Decision making on the postnatal ward
	Repetition of BCG information and vaccine offer: a welcomed reminder
	What is BCG?
3. Parental concerns specific to the BCG vaccine	Proximity to primary immunisations
	Age of baby
	Fever and wound healing
	Selective immunisation programme
4. Getting a BCG appointment	Parents bounced between services
	No invite, no vaccine
	What is SCID screening?
5. Attending the appointment	Familiarity and proximity of vaccine clinic location
	Transport - Car vs. public transport
	Financial Costs

### Eligibility assessment gone wrong: incorrect referrals, confusion, and parental frustration

#### Assessment errors

Parents reported their babies were assessed for neonatal BCG eligibility on the postnatal ward, with some health visitors also checking during postnatal visits. Eligibility assessment is when most interviewees first found out about the BCG vaccine. From the 30 parents interviewed, five ineligible infants were invited for BCG. Assumptions were made about their country of birth having a high prevalence of TB leaving parents confused about why they were invited.

“We asked the nurse when we came to the appointment, and she wasn’t really sure. She said, “I don’t see anything in the letter, but if you want to take it, you’re here,” and so we just took it. I did not ask about eligibility to the midwives in the antenatal period before birth or that in my mind I was not even and Albania, I think, was one of the lowest for Europe, and Lithuania, as well.” (UA1.02 vaccine on time).

Parents also reported experiences of the vaccine being offered contrary to the BCG protocol, which could create inequity in the immunisation programme.

“…we accepted all. They say as you like, you can accept vaccine, and you can say no. Iraq is not any more in high risk. Iraq is not in the list for TB, because we were originally from Iraq, so they say in the hospital, they say we check it, your country back home is not in the list or like it’s a country from Africa, they have a high risk. So, as you like, but we advise you, your child must be taking this vaccine. Me and baby mum, we signed yes of course…” (UA2.09 vaccine on time).

There were a few examples where parents had been told on the postnatal ward that they were eligible, when they were not, and subsequently did not receive an appointment letter. This caused distress to the parents who thought their baby was at risk, were waiting for an appointment to come through, and chasing HCWs for an appointment, which did not come.

“… the doctor or the nurse said that, yeah, he would be eligible for BCG, and they will do a referral to the practice, to the GP. We didn’t receive anything from the GP…when my health visitor came…she asked where we were born and then she had a leaflet so she checked and she said he would be eligible for BCG, because both of you are from Egypt. And I went to health visiting drop-in for I think three, four times and every time I mentioned that I’m still waiting for the BCG appointment. And two times they said they will chase it and until now, I just didn’t. He will be four months in a couple of days… I don’t know who is responsible. I mean I don’t know who to contact…” (UA2.06 unvaccinated).

Another administrative error within the sample included an example of a parent being “*shocked*” their ethnicity was recorded incorrectly “*I told them Indian*,* but they ended up writing down Pakistani”* (UA2.10 unvaccinated).

#### Parent understanding of risk and eligibility

Parental understanding of risk, and the reasons for eligibility were mixed. Some felt it was because they had “*more of an affinity to get to TB if…exposed”* (UA2.11 vaccine delayed) due to their ethnicity, while others thought it was only important because of foreign travel.

“…we called and cancelled the appointment, just because we thought well based on everything we read, it’s not really necessary at the moment. Like I haven’t been to India for a few years, and I didn’t think we would want to go with the baby, but we said at that point, we’ll just plan this, and we’ll get the vaccine at that point, like however long before the trip was necessary.” (UA2.10 unvaccinated).

“Maybe the criteria was people with dark skin…” (UA2.06 unvaccinated).

### How BCG information is provided by the health service

#### The ‘*surprise*’ vaccine

The neonatal BCG vaccine was referred to as ‘the surprise vaccine’ which captured interviewees common experience of a lack of discussion about BCG vaccination during any antenatal appointments. Most interviewees found BCG vaccine was first raised on the postnatal ward and was seen as *“…different because that was presented as kind of a surprise vaccine*,* probably because we didn’t know about it before…”* (UA2.10 unvaccinated). Parents felt this absence of information during antenatal period was a missed opportunity.

“…perhaps giving the information about vaccinations antenatally rather than, “You’ve had a baby. Here’s a load of leaflets,” and “Are you opting in for things?” giving parents the opportunity, I guess, to educate themselves beforehand and understand why it’s important.” (UA2.02 vaccinated on time).

Even when infant vaccines were raised by the parent with a health care worker during the antenatal period opportunities to discuss the BCG vaccination were missed.

“I even told my midwife I was planning to fly to Brazil (to see family)…traveling with him with two months…And she just suggested have the first vaccines, like eight weeks, and then that should be fine. But we never talked specifically about any specific vaccine.” (UA1.10 vaccinated on time).

We only found one opposing case where a participant thought they remembered the midwife talking about immunisations for their baby, however they were not certain.

“I think the midwife mentioned it a little bit during pregnancy as well but didn’t give any leaflets or anything.” (UA2.08 vaccinated on time).

#### Decision making on the postnatal ward

Due to the reported lack of information on BCG antenatally, information on this ‘surprise vaccine’ was mainly first received on the postnatal ward. A strong theme that emerged, especially from first-time parents, was this was a difficult time and place to receive new information and decide about vaccination. Additional complexity postnatally, for example if the baby was premature or unwell, exacerbated decision making.

“…when she mentioned it initially, my head was all … I just was completely drugged up on whatever pain, the codeine…Post-surgery, I was just in a complete daze, so I hadn’t actually had time to think about it…Because I just think that post-birth couple of days, I was not in my right mind. I was in so much pain and very hazy. It’s not a good decision-making frame of mind.” (UA1.03 vaccine delayed).

“I was thinking, “I’ve just given birth. I don’t think I’m really in a position to think about that thoughtfully.” (UA1.07 vaccine delayed).

“….I had so much going on. I was sleep deprived, people coming in and out of my cubicle trying to do observations and so many things every few minutes. And I was trying to feed the babies, desperately, because feeding was a really key issue that was leading to complications. So, when it was just another person coming in with a form being like, “Here, we need to ask you some questions” it’s sort of like you only have a minute to make the decision…it’s not really the best time to be carefully considering things and making decisions I guess” (UA1.15 unvaccinated).

#### Repetition of BCG information and vaccine offer: a welcomed reminder

What helped parents with accessing the vaccine was repetition of BCG information at different stages. For example, more than one health professional checking parents had received information about BCG vaccination, especially outside of the postnatal ward.

“Yes, no, the health visitor did. Yes, I remember now, she came maybe day 10 or something like that, maybe a bit more, and she said, “Have you had the vaccine?” I said, “No.” And then she explained to me…” (UA1.16 vaccine delayed).

Repeated information about BCG vaccination can help parents change their minds. For example, because the vaccine was offered to them at a stressful time, one mother declined vaccination and was discharged from the pathway (as per neonatal BCG protocol [21]). However, during the interview she stated she would like to get her twins vaccinated.

“They’re nine months…they were offered it when I was in hospital after their birth and I said no. And then later I found out more about it and decided that actually I would’ve wanted to get it.” (UA1.15 unvaccinated).

#### What is BCG?

Being presented with an unfamiliar name for the vaccine was confusing *as “there were just a lot of acronyms”* (UA1.09 vaccinated on time). Some parents were familiar with the term BCG, having had the vaccine themselves or because they were aware of BCG vaccination programmes in their nation of birth. Others found the term ‘BCG’ very unfamiliar and did not associate it with preventing TB disease.

“…like it was just an acronym that I hadn’t heard of, and I wasn’t really aware…I was just in a frame of mind of saying no to any stuff that seemed a little bit like an extra test or hospital thing in that whole context.” (UA1.15 unvaccinated).

Some parents had concerns and questions about the reason for BCG vaccination and asked for more information to help support their decision. Requests for further information were not always within the knowledge base of healthcare workers who then referred parents to the internet.

“…she wasn’t actually sure what the details of it were, but she said to just check it out on Google and make sure we were all happy with it. But we didn’t really get any information on why it was being offered to us specifically.” (UA2.10 unvaccinated).

The absence of information provision by healthcare workers often resulted in parents researching the BCG vaccine online (sometimes this was prompted by the healthcare worker themselves). For one parent, this self-directed research resulted in non-vaccination.

“So that’s why when I was doing this research, finding out all the cons, finding out about the side-effects and stuff, that was enough for me to stop and go, “OK, no, I don’t want to proceed with this… we called and cancelled the appointment” (UA2.10 unvaccinated).

### Parental concerns specific to the BCG vaccine

Many parents in our sample were keen to vaccinate their children to protect them from infectious disease as early as possible. Some parents had specific concerns about the BCG vaccine. These included the age of their baby at time of vaccination, anxieties about side effects such as fever and the potential for a wound at the injection site.

#### Proximity to primary immunisations

There were mixed feelings about the proximity of BCG vaccination to the primary immunisations given at 8 weeks. Concerns varied and depended on how close their BCG appointment was to the 8-week vaccinations. Some participants trusted their nurses and doctors’ reassurance that it was safe whereas others opted to delay the primary immunisations for a bigger gap, *“…they are quite close*,* so that’s the reason why I did delay his immunisations a little bit*,* not too much*,* just two weeks…”* (UA1.04 vaccine delayed). More information on safety of BCG vaccine being close to primary immunisations would have been helpful for some participants.

“I asked the BCG people that there will be – “Shall I go for the normal vaccination the next day?” The answer that I’m given is, “It’s your child. We don’t have any guidelines.” If you ask the person giving the first vaccination “We did the BCG yesterday, is it OK to go?” “It’s your child, we don’t have any guidance.” That’s kind of a crap answer.” (UA1.11 vaccine delayed).

#### Age of baby

Parents views were mixed about the age of the baby when they received their BCG vaccine. Several parents were happy for their baby to be vaccinated as soon as possible for *“fear of getting him exposed to pathogens”* (UA1.02 vaccinated on time) or because *“while they’re young*,* just because it’s easier”* (UA1.04 vaccine delayed). Others thought, *“Oh gosh*,* that’s so young to have a vaccine”* (UA2.08 vaccinated on time) or *“…so new*,* so fresh*,* that we didn’t really want him jabbed with a needle”* (UA2.10 unvaccinated) which, for some, resulted in vaccination being delayed until babies were older.

“…delayed it for the first appointment just because I thought they’re really young, and I just thought they’re a bit – you know, they’re tiny, their tiny arms and things, so yes, I did delay it for when they’re a bit older.” (UA1.16 vaccine delayed).

#### Fever and wound healing

Concerns about babies having fever post BGC vaccination were due to the age of the child, “*The one that worried us most was the fever…just the thought of the baby having a fever when at the time you can’t give him Calpol”* (UA2.10 unvaccinated). Parents who attended appointments and asked about fever, found the nurses *“reassured my worries”* (UA1.05 vaccinated on time).

A few parents voiced concerns about wound healing associated with BCG vaccination, “*I was a bit concerned*,* you know*,* because some of them can get like the open puss-ie*,* wound on their arm and I was worried that…” (UA2.08 vaccinated on time).*

#### Selective immunisation programmes

Some parents spent more time deliberating on selective immunisation programmes than universal programmes. They viewed BCG as an extra one and were more cautious about accepting it and wanted more information about it compared with universal vaccinations.

“…our standpoint on vaccines is if they’re standard then everybody always gets them unless you opt out, then we’re happy to do it. If they’re an extra one then we think a little more carefully about it.” (UA2.11 vaccine delayed).

### Getting a BCG appointment

#### Parents bounced between services

Some participants found the neonatal BCG system frustrating and discussed being bounced from one health professional to another to get a referral or to be able to make an appointment for vaccination.

“So, when I got the leaflet, this leaflet was saying I should contact my GP, my midwife or health visitor to get the vaccine, to get access to the vaccine. So, I asked my GP. He sent me to the midwife. I asked my midwife, she sent me to the health visitor. I asked my health visitor, she sent me to the GP, you know? So, this was a horrible case.” (UA1.10 vaccinated on time).

“So, trying to get a hold of that when you are going from GP to health care to midwife to this person to that person until you finally – it took many months to finally find, to be able to call that number to then access what we needed.” (UA1.12 vaccine delayed).

Parents in contact with a healthcare worker who knew the system, found it easier access to the vaccine, *“the health visitor who came to visit us here*,* said*,* “Oh yes*,* I’ll sort that out*,*” and then she sorted it.”* (UA2.08 vaccinated on time).

#### No invite, no vaccine

Parents were notified of the vaccination appointment via letter, text or phone call but often this arrived at short notice or was a surprise to parents.

“…getting the letter a day before wasn’t great. It just set me in a very anxious mode because I was like, what if it’s too late to cancel?” (UA2.13 vaccine delayed).

“…the text did come out of the blue. The invitation or the appointment text came out of the blue without, yeah, warning.” (UA1.08 vaccine delayed).

Some parents reported that they were either not notified of appointments or that notifications were different to other ante/postnatal appointments.

“…it was a little bit after the time that he should…it was at five months he was given it …I got referred for it and then I didn’t hear for a while and then I got a phone call to say, “Just wanted to confirm that you’ve got an appointment to do your vaccine on Friday,” and I said, “Which vaccine?” …You should have got a letter.” And I said, “Oh, I haven’t got any letters.” But the letter then arrived, actually, three days later in the post. They arrived after they rang me…it was supposed to happen, but I didn’t know.” (UA1.07 vaccine delayed).

“…the other vaccinations…would come up on the app, so I kept checking and checking and checking and then, when it didn’t, that’s when I was a bit confused. And it was only when I got the letter that I actually knew the time” (UA2.14 vaccinated on time).

#### What is SCID screening?

Almost all parents had not heard about SCID screening. A couple had heard of SCID screening but were unaware this was the reason for resulting time change for neonatal BCG vaccine. Some parents noticed a difference in the delivery model if they had older children or were familiar with neonatal BCG programmes in their country of birth.

“Well, I did notice it because I think the health visitor that came asked if he’d had the vaccine or if I had the appointment. So, I did ask her, “Oh they usually give it at the hospital, but they didn’t, and she just explained that they delay it now.” (UA2.05 vaccinated on time).

“…back home in India, the vaccination is given pretty early on. It’s almost given when you I think are not two days old. Like, here it’s a bit more relaxed” (UA1.11 vaccine delayed).

Researchers explained the rationale for the programme change during the interviews. Almost all parents felt the change was a good idea, even if it meant receiving the vaccine was more inconvenient for them. A couple of parents felt it depended, *“if it makes it less accessible for people to get the vaccine that’s something you want to weigh up against the…pros and cons”* (UA1.15 unvaccinated).

“I think I would have been happy for her to have it straight afterwards. It’s almost the logistics around sort of getting a four-week-old out and myself out of the house, and because I had an episiotomy.” (UA1.08 vaccine delayed).

Some parents found the wait for the vaccination stressful and would have liked communication about the timing change or schedule in England.

“Nobody told us anything about SCID linkage to BCG. You are the first person telling me that…I totally agree with that if there is a reasoning why not, but would be nice to be informed because system breaks.” (UA1.11 vaccine delayed).

### Attending the appointment

The location of the neonatal BCG vaccination clinics differed between the two urban areas. In one area, the parents were asked to attend a community venue in the local authority area they lived. In second area parents were asked to return to the hospital their baby was born. For most participants there was only one place offered, and in the first urban area often somewhere unfamiliar to them.

Views on how easy it was to access BCG clinics diverged. This was due to several factors, which included access to a car, distance to appointment, the place the vaccine clinic was hosted, and whether there was additional support (e.g. partner, car) to help them attend the appointment. Most parents stated that the BCG appointment was the first time they left the house with their baby since birth and relayed that the journey could be complicated by factors associated with both vaginal and c-section birth recovery.

#### Familiarity and proximity of vaccine clinic location

In urban area 1, where some parents were offered a local location (e.g. hosted in their local GP surgery), this was “*quite straightforward*,* really easy”* (UA1.04 vaccine delay) or *“literally down the road…quite convenient”* (UA1. 05 vaccinated on time). However, most parents reported significant travel distance and or time, either returning to the hospital of birth (urban area 2) or to a community location such as a children’s centre (urban area 1). Resultantly, a recurrent concept was that parent’s would prefer the vaccination to take place somewhere close and familiar to them.

“It takes us about 45 minutes from where we currently live… that was the only location that we were offered.” (UA2.08 vaccinated on time).

“…it was quite a random, far place to go. So, I’ve never been to that centre before, and we’ve never been since, so maybe having one location that I’m familiar with or that we’re going to be going to again would have been helpful, because it was a bit of a mission…I mean, it just required a bit more planning…“Oh, God, it’s all the way over there…” (UA1.08 vaccine delayed).

“…get the GP to do it rather than having us travel to a different centre altogether. Why wouldn’t the GP do a BCG? Didn’t get that part. And it’s far… if all the vaccination done at the GP then why not BCG? And I’m not sure what’s different?” (UA1.11 vaccine delayed).

Some participants would have preferred a clinical or home setting as they felt that would be safer and cleaner for their baby, while others were happy to receive the vaccine in a community venue.

“…it (children’s centre) wasn’t the one next to me, it was further away, but I mean it wasn’t too bad. And they had a car park as well…so that was all right. I drove my car there. I had a better experience, they weren’t rushing me to get out which is – the GP surgery they’re like “Yes, can you get in and out,” kind of thing.” (UA1.16 vaccine delay).

“I think it was a community area inside the church. It didn’t really feel clinical…It was a table in this area where they probably do kid’s birthday parties. It didn’t feel super-clean…obviously, the nurses had the disinfection. For me, I would have taken it in the parking lot of a supermarket…or the schoolyard, or whatever. But for him…a lot more on the too careful side of things” (UA1.02 vaccinated on time).

#### Transport - car access vs. public transport

Car access was repeatedly mentioned as an important enabler to attend the vaccination, especially considering mothers were still recovering from birth and looking after newborns.

“We drove there, and it was the first car trip that we’ve been on with the baby…And to be honest, if we didn’t have the car I don’t actually know how we would’ve gotten there” (UA1.9 vaccinated on time).

“I had had an episiotomy, I wasn’t walking large distances. We have a car…thankfully.” (UA1.8 vaccine delay).

Some parents without a car, considered or resorted to hiring one because of the proximity of the appointment to birth and their recovery, “*We end up going by car*,* so we rented a Zipcar. It’s mainly because of my discomfort. So*,* after birth I had a bit of pain”* (UA1.10 vaccinated on time).

For those who had to or would have to rely on public transport, this was a challenge, especially for a longer distance *“I need to take about two buses*,* it was not really easy*,* but I didn’t have a choice… it was about one hour…”* (UA1.13 vaccinated on time).

“Yes, so travelling with the babies is quite difficult by myself because it’s hard to handle if they both get grumpy or needy and I’m on the bus or something without an extra helper there…if I had to wait around for the appointment and then the babies get grumpy…they’re getting a bit too heavy for me to go very far at the moment with that. Yes, it limits me. It limits how far I can go from home basically.” (UA1.15 unvaccinated).

Social support was reported as being helpful and, in some cases, crucial to overcoming the travel distance, transportation, recovery from birth and support with older children.

“…the other good thing is that he was home for paternity leave, so because the location for the vaccine was a little bit further out, so about 40 minutes on the bus… Because I’ve got that support as well, and that’s why it was a lot easier for me.” (UA1.4 vaccine delay).

“It takes us about 45 minutes from where we currently live…It was fine…my husband drove and so he had paternity leave so that was lucky that, you know, it was within the ten days that he could come and do the driving and help with the kids.” (UA2.8 vaccinated on time).

#### Financial costs

While car access and social support were enablers to attending the appointment, these enablers came at additional costs for some parents.

“…it stressful obviously because I need to pay for the parking. For me, I was upset as well and for me, it’s hard for me to pay….my partner he had to take off…he didn’t get paid. Whenever he like gets a day off from them, they never pay him the money.” (UA2.04 vaccinated on time).

## Discussion

This study explored parents’ experience of the new neonatal BCG vaccination pathway in England. While parents were unaware of SCID screening and the resulting changes to the BCG pathway, most participants were accepting of the rationale for the new BCG pathway (provided service delivery could be improved). This mirrors findings in the primary service evaluation [16] that reported vaccine providers found parents were unaware of SCID, which meant they had to explain the changes to BCG vaccination provision. Here we highlight key findings and contrast these with the previous BCG service evaluation (from the perspective of commissioners and providers) [16] and the wider literature. Areas of consideration for policy and practice are presented.

In the preceding service evaluation [16], increased DNA rates due to the loss of a captive audience and difficulties in meeting the 28-day vaccination target were key concerns which were echoed in the literature [12, 13]. Many of the reasons suspected as affecting uptake such as clinic suitability, concerns regarding the babies age, side effects, proximity to the routine 8-week immunisations, and a desire for more time to decide were all strongly supported by parents within our sample. While commissioners and providers reported a need for improved BCG education and literacy, the absence of information provision and resultant misunderstandings of risk and eligibility is concerning.

### When BCG is first discussed with parents

This study reveals that neonatal BCG vaccination is often sprung upon parents directly after birth with little warning and a limited time for decision-making. This has the potential to result in vaccine refusal and discharge from the BCG pathway, despite parents being open to vaccination after the delivery period. Having left the pathway, it can be difficult for parents to secure BCG appointments and are often ‘bounced’ between the General Practitioner (GP) and other healthcare practitioners.

The lack of antenatal discussion about BCG vaccination reported by parents is a missed opportunity. In England, the NHS and health professionals are the most trust sources of information on vaccination [22]. Parents often make decisions about childhood vaccinations during pregnancy [23]. Midwives, as trusted health professionals, are a well placed to provide information on the neonatal BCG programme, as well as vaccines in pregnancy, to expectant parents. However, midwives state time pressures and inadequate education on immunisations as barriers to effective communication with families about vaccinations [24]. Midwives also reported being unconfident in answering parents’ questions about vaccinations [24]. Our results support previous literature on the importance of the antenatal period for early discussion around childhood vaccination, especially for vaccinations provided to babies soon after birth. To achieve this, midwives need appropriate education and support to have these conversations.

### Eligibility assessment

A minority of commissioners and providers also raised concerns regarding both missed and inappropriate BCG referrals [16]. This was attributed to BCG eligibility being an optional field in the data system, with suggestions of turning this into a mandatory item for form submission. Our study with parents confirms these concerns. Despite a limited sample size, we spontaneously encountered numerous parents who had either received BCG vaccination despite not being eligible, or who had been wrongly informed of eligibility (sometimes on numerous occasions). The sense of unfairness, stress, and confusion this can cause for parents is a novel contribution of this study and provides a different perspective to the problem as experienced by parents. This contradicts the BCG patient flow chart [21] which states that BCG eligibility should be identified and recorded during the maternity period further highlighting the absence of information provision during the antenatal period.

Accurate assessment is at the heart of selective programmes, ensuring that babies at risk of exposure to TB are offered BCG vaccination. Furthermore, it is required to provide a reliable denominator for monitoring progress in meeting the TB action plan targets [14]. It also allows parents to make informed choices about vaccination based on risk of exposure to TB and risks associated with vaccination, prevents concerns raised by inaccurate eligibility assessment, and increases parents’ confidence in health care providers and the recording of their data. Accurate eligibility assessment also ensures appropriate use of public funds based on provision of vaccines for those most at risk.

### Novel barriers to neonatal BCG vaccination in the new model of delivery

Beyond these, however, there are key barriers to vaccination faced by parents which were not considered by commissioners and providers which deeply shaped parents experiences:

- Firstly, the proximity of the vaccination to birth (2–4 weeks postpartum), the physical discomfort of travel for mothers, and the resultant reliance on social support and easy transportation (ideally a car). These enablers were not available to all parents and came at an additional cost for some. While there are other appointments a mother may need to attend outside of the home soon after delivery, this paired with the long distance to clinics (up to one-hour each way) made attending BCG vaccination appointments painful and physically challenging. Empirical literature cites the importance of continuity of healthcare facilities in facilitating positive experiences, engendering trust, and improving uptake among migrant groups [25, 26]. This requires attention given the 44% of providers were yet to complete an accessibility assessment and 57% reported travel distance for parents up to a maximum of 10 miles [16].
- Secondly, the interpretation of the BCG vaccine as an ‘extra’ or an ‘optional’ vaccine given its representation as a ‘selective’ vaccine. This is an important insight that highlights the need to consider how representation of the programme as ‘selective’ may unintentionally affect parents’ perception of the BCG vaccine.
- Thirdly, the lack of awareness among GPs and other community healthcare practitioners on how to refer parents for BCG vaccination.
- Finally, missing appointment notifications, despite commissioners and providers feeling more confident in their appointment and data systems at the point of interview.

### Separateness of the BCG service

Our interpretation is that the barriers parents faced (e.g., distant clinic locations which do not conform to GP delivery, limited healthcare worker knowledge of the BCG vaccine and referral pathways, heightened decision-making, different notification methods) relate to the separateness of the BCG service from other familiar vaccination, postnatal, or primary care services. Notably, the neonatal BCG service is commissioned and delivered separately to other infant vaccinations. This is because the BCG vaccine is delivered via the intradermal route, which is different from many routine immunisations and requires suitable training which may not be held by a parent’s GP [1, 27]. While the funding structures and requirement for specialist trained BCG immunisation nurses [27] add complexity, our data suggest the current delivery model is causing notable barriers to vaccination. Efforts are needed to try and achieve a more harmonious interface between the selective and routine programme (as experienced by parents) to support uptake of the BCG vaccine.

We recognise implementing the new pathway was challenging for commissioners and providers, and that issues with insufficient staffing and resourcing are ongoing [16]. While some commissioners and providers reported bespoke initiatives to improve coverage these were often delivered by teams with additional budgetary and administrative support [16]. Rather than a critique of those working to deliver the service under difficult circumstances, we hope that these findings will enable targeted intervention and successful utilisation of the resources which are available, while representing the potential coverage gain which could be achieved with greater investment.

For parents with babies eligible for neonatal BCG, this is likely to be their first experience with childhood vaccination. How they experience this BCG pathway could impact how they view vaccinations, and could impact subsequent childhood vaccination uptake.

### Areas of consideration for policy and practice

1. Consider revision of term ‘selective vaccination’ in communication resources to ensure parents understand eligibility is based on risk.
2. Promote adoption of existing childhood vaccination and BCG resources during antenatal appointments and ensure midwifery staff have the training, resources, and time to do so.
3. Ensure eligibility assessment is carried out according to protocol to ensure eligibility is correctly recorded and communicated to parents – this includes emphasising that simply because the vaccine is ‘selectively’ offered that it is still essential for their baby’s protection (and why).
4. Support healthcare professionals’ (especially primary care and health visiting) understanding of the neonatal BCG programme and referral pathway to: prevent ‘bouncing’ parents around; act as a failsafe for missing appointment notifications; and to issue a repeated BCG offer for parents who may have changed their mind.
5. Improve timing of appointment notifications in proximity to vaccination date to allow new parents to have time to prepare or rearrange appointments.
6. Review location of vaccination clinics – consideration should be given to how far parents must travel and if the location is easily accessed via public transport, particularly given the context of mothers’ birth recovery and the increased costs associated with car hire or long journeys.
7. In the English context, to reflect on how the neonatal BCG service may be better integrated with other community, postnatal systems.

### Strengths and limitations

Our results were shared with professionals (including clinicians) working in the subject areas, for the purposes of expert validation. These individuals, who have direct experience with the phenomenon under investigation, confirmed that our themes and interpretations reflected their own experiences. Using a multidisciplinary research team (including psychology, nursing, anthropology, public health, and medicine) enabled diverse interpretive lenses. We used reflexivity and triangulation between the team to support reliability of results.

As a qualitative investigation this study provides further insight and novel understanding of barriers to uptake of the BCG vaccination from the perspective of parents. This provides added value to the provider and commissioner evaluation [16] which focused on programme implementation. Despite the richness of the dataset, our findings may disproportionately reflect challenges or perspectives within the two urban areas where the research took place. Furthermore, while recruitment of those with mixed eligibility and uptake status is a strength, it was not feasible to identify or include parents of eligible babies who had been missed during assessment. While this would have demonstrated limited access to services in its most extreme form, we would have been unable to speak to parents about their experiences of interacting with the service given this had failed to materialise and was therefore beyond the scope of this evaluation. While efforts were made to engage parents excluded from healthcare services via community centres, this still reflects a level of community participation. Moreover, while some participants had basic level of English or English was not their first language, no participants included in this study required translation services for the interviews. This means some specific barriers to accessing the vaccination for parents who do not speak English may not be included in the results and findings. This inequality was a specific concern raised by some paediatricians and providers about the pathway change [12, 16]. We received feedback from a couple of community groups that new migrants with limited English would not be comfortable with one-to-one interviews. They recommended focus groups co-facilitated with a trusted member of their community to gain insights into parents’ experiences. This data collection approach was omitted in the ethics committee approved protocol and time restrictions meant we could not submit an amendment. Future evidence gathering opportunities in this field needs to be mindful of this recommendation.

## Conclusion

Prior to participating in this study, parents were unaware of SCID screening and change to the neonatal BCG vaccine schedule. Parents were generally accepting of the rationale for the new pathway; however, we identified distinct accessibility concerns that varied from those associated with the routine immunisation programme. These barriers, and the separateness of the BCG programme from routine services, impacted parental experiences and vaccine uptake. For a selective vaccination programme this may also result in inequalities in uptake.

The neonatal BCG remains an important component of TB prevention in England. Currently the way it organised adds complications for parents accessing the vaccine for their baby. Those involved in the delivery of BCG vaccination should be allocated resources to reduce barriers to neonatal BCG vaccination.

## Supplementary Information

Supplementary Material 1.

## Data Availability

The datasets generated and/or analysed during the current study are not publicly available due as they contain information that could compromise the privacy of research participants but are available from the corresponding author on reasonable request.

## Abbreviations

BCG: Bacillus Calmette-Guérin
DNA: Did not attend
GP: General Practitioner
LSHTM: London School of Hygiene and Tropical Medicine
JCVI: Joint Committee for Vaccination and Immunisation
NHS: National Health Service
SCID: Severe Combined Immunodeficiency
SSI: Semi-structured interviews
TB: Tuberculosis
UKHSA: UK Health Security Agency
WHO: World Health Organisation
WNB: Was not brought

## Footnotes

1 Providers and Commissioners [16] referred to DNA so we have used this term. We’re aware the phase Was Not Brought (WNB) is used more commonly in paediatric services.
