# Supplementary material for: Parents’ perspectives of the new neonatal BCG vaccination pathway in England: a qualitative study
Source: BMC Public Health. 2025 Aug 18;25:2821. doi: 10.1186/s12889-025-23859-x (PMC12359992; doi:10.1186/s12889-025-23859-x)
Supplement: Supplementary file 1 — Supplementary Material 1. [file 12889_2025_23859_MOESM1_ESM.docx]

**Supplementary Table S1 - Topic Guide**

| **Background information** | 1. Is this your first child? 2. (*If no)* Can you tell me a bit about your other children? Were they offered the BCG vaccine; did they receive it?    1. *Why/why not?* |
| --- | --- |
| **Awareness** | 1. What information have you received about BCG vaccination? What did you think of this information?    1. *How was it shared (e.g., verbal, flyer, web link)?*    2. *How easy/difficult it was to understand?*    3. *How trustworthy it was? Do you feel you have enough information on the benefits/risks of BCG vaccination?* 2. What do you know about the BGC vaccination?    1. *What does it protect against?*    2. *Who is eligible (I.e., the fact it is a selective programme)? Do you know why you were offered the vaccine? Were you asked by a health professional any questions about travel or visiting relatives/or are you aware you live in an area where everyone is offered the vaccine?*    3. *When it is due (i.e., 28 days)?* |
| **Access** | 1. Has your child received the BCG vaccination or not? Why is this?    1. *Psychological factors (i.e., decided not to vaccinate)?*    2. *Physical factors (e.g., lack of appointments, etc.)?*    3. *Reasons for vaccination* 2. (If applicable) Tell me about your experience of trying to access the BCG vaccination for your child?    1. *Experience of appointment booking systems?*    2. *Any language barriers?*    3. *Location of vaccination services/travel distance/mode of transport?*    4. *Appointment times?* |
| **Affordability** | 1. How does the cost of the BCG vaccination (for example, travel costs, lost earnings, etc.) affect your experience/decisions regarding BCG vaccination?    1. *Not only financial costs but time costs?* |
| **Acceptance** | 1. How do you think/feel about the BCG vaccine?    1. *Side effects post vaccination?*    2. *Safety?*    3. *How well it works?*    4. *Proximity with other routine vaccinations?*    5. *Age of child?* 2. How do you think/feel about the disease the vaccine protects against, tuberculosis (aka. TB)?    1. *Seriousness?*    2. *How common do you think it is?* 3. How do you feel about vaccination generally?    1. *Trust?*    2. *Have you vaccinated your child(ren) for other diseases?* 4. How do your friends (or healthcare workers) feel about BCG vaccination? How does this affect how you feel towards the BCG vaccine? 5. Do you feel a social responsibility to vaccinate your child against BCG or do you feel as a parent that is your personal decision? 6. Are you aware of the recent change to the BCG vaccine from being given at birth to being given at day 28? OR (If not first child) did you notice the change in vaccine due date? 7. Does SCID or severe combined immunodeficiency sound familiar to you? It is part of the newborn blood spot test (via heel prick) when baby is 5 days old? Do you remember hearing anything about this?...[wait for initial participant response]...The date of vaccination was changed in 2021 due to the introduction of screening for this.    1. *What do you think about the change to the BCG immunisation date due to SCID screening? Do you think this is a good/bad idea?*       1. *In terms of period of no protection for baby against TB?*       2. *In terms of additional protection against negative BCG reaction?*       3. *In terms of additional appointment to infant vaccination schedule?* |
| **Activation** | 1. Did you receive any prompts/reminders about the BCG vaccination? |
| **Reflective questions** | 1. What are the largest barriers in accessing the BCG vaccination for your child? 2. What could make this better? 3. What are the largest enablers in helping you access BCG vaccination for your child? 4. Do you feel that your circumstances in any way shape your ability to understand, reach, or use the BCG vaccination service?    1. *Occupation/employment?*    2. *Educational/language background?*    3. *Degree of family support?*    4. *(If yes), how do these circumstances affect your ability? Is there a solution?*    5. *Heritage/ethnicity (experience of vaccination in other countries)*    6. *Age of parent*    7. *Other children* 5. Is there anything you feel is important which we have not discussed? |
| **Final checks** | Can I quickly ask how you heard of the study? |
| *Post-interview checklist* | - Audio-recording handling.   - Ensure recordings of the interview are saved.   - From the main device upload the audio-recording to the secure OneDrive.   - Log out/in and check that the audio-recordings have successfully been uploaded onto the drive.   - Delete the audio-recordings from the data capture device.   - Update data register on the One Drive. |
